# Knowledge of Periodontal Diseases, Oral Hygiene Practices, and Self-Reported Periodontal Problems among Pregnant Women and Postnatal Mothers Attending Reproductive and Child Health Clinics in Rural Zambia

**DOI:** 10.1155/2018/9782092

**Published:** 2018-08-07

**Authors:** T. M. Kabali, E. G. Mumghamba

**Affiliations:** ^1^Department of Orthodontics, Paedodontics and Community Dentistry, Muhimbili University of Health and Allied Sciences (MUHAS), P.O. Box 65014, Dar-es-Salaam, Tanzania; ^2^Department of Restorative Dentistry, School of Dentistry, Muhimbili University of Health and Allied Sciences (MUHAS), P.O. Box 65014, Dar-es-Salaam, Tanzania

## Abstract

**Aim:**

To determine the level of knowledge of periodontal diseases, practices regarding oral hygiene, and self-perceived periodontal problems among pregnant and postnatal women attending reproductive and child health clinics in rural districts of Zambia.

**Methodology:**

This was a quantitative, questionnaire-based, descriptive, and cross-sectional study that recruited 410 women aged 15 to 43 years. Data were analyzed using SPSS v19.0 computer program.

**Results:**

Participants knowledgeable of periodontal diseases were 62%; gingivitis signs included gum swelling (87.4%) and bleeding (93.3%). Of all participants, 95.6% practiced tooth brushing: twice/day (38.5%), using plastic toothbrush (95.6%), chewing stick (12.2%), toothpick (10.7%), dental floss (2.0%), and tongue cleaning (55.4%). Self-reported periodontal problems were bleeding gums (23.2%), gums that were reddish (10.5%), swollen (11.0%), painful (15.9%), and mobile teeth (3.4%). In logistic regression analysis, painful gums, reddish gums, and toothpick use were 21.9, 4.7, and 4.3 respectively, significantly more likely to cause gum bleeding on tooth brushing.

**Conclusions:**

Most studied women had general knowledge of periodontal diseases but only few knew the cause. All participants performed tooth cleaning; however, majority did not know appropriate practices, and only few had periodontal problems. Integration of oral health to general health promotion and periodontal therapy to pregnant women at high risk is recommended.

## 1. Introduction

“Periodontal problems” encompass several conditions that include gingival and periodontal diseases [1]. Periodontal diseases always start as gingivitis which denotes an inflammation of gingival tissue due to microbial challenge [2]. Epidemiological studies show that gingivitis may or may not progress to periodontal disease [3]. However, the most significant factors for gingivitis to progress to periodontal diseases include presence of periodontal pathogens in particular the red complex that includes the *Porphyromonas gingivalis*, *Tannerella forsythensis*, *Treponema denticola*, genetics, and poor oral hygiene in the form of accumulation of oral biofilm that is full of millions of microbes (1 gm contains more than 10^11^ microorganisms) and of different types as gram positive and gram negative [4].

During pregnancy, periodontal tissues' response to biofilm challenge is reinforced as female sex hormones are necessary but not sufficient to produce gingival changes by themselves and usually plaque plays a role [5]. Pregnancy period is accompanied by an increase in the levels of both progesterone and estrogen which by the third trimester, reaches levels 10–30 times more than the one seen during typical menstrual cycle, and changes in the gingiva include an increase in gingivitis that usually starts during the second to third month of pregnancy and increases in severity through the eighth month, where it decreases along with the abrupt decrease in hormone secretion [6, 7]. Pregnancy affects the severity of previously inflamed gingival tissues but does not alter healthy gingiva [8]. Pregnant women with previous chronic gingivitis which attracted no attention before pregnancy become aware of their gingival status as the previously inflamed areas become enlarged and edematous and more noticeably discolored with an increased tendency to bleeding [9].

An intensive approach to plaque removal may be effective to treat pregnancy gingivitis and all forms of gingival enlargements [10]. Improving maternal oral hygiene is important for oral health and may reduce systemic proinflammatory cytokines and improve maternal outcomes [11]. In a study involving 409 postpartum women, only half of the women brushed their teeth more than once a day for about 1–3 minutes; in addition, a substantial proportion of patients (35%) reported seeking oral care from a dentist only when they experience pain, thus making preventive strategies less possible [12].

The impact of periodontal disease on pregnancy outcome is now under scrutiny. Findings from observational studies yielded inconsistent conclusions on the relationship between periodontal disease and various pregnancy outcomes (including early pregnancy loss, preterm birth, low birth weight, and preeclampsia) [13]. A close relationship was shown to exist between lack of oral hygiene and periodontal disease in pregnant women [14]. Most of these ill-effects could be avoided by good oral hygiene practices [15]. Traditionally, tooth brushing using manual or powered toothbrush and flossing have been considered the standard for routine plaque removal and gingivitis reduction [16]. In Zambia, retrievable information on oral hygiene practices and gum/periodontal problems among females at reproductive age in particular pregnant women and mothers for young children is lacking. The aim of this study is to assess knowledge on periodontal diseases, practices regarding oral hygiene, and self-reported periodontal problems among pregnant women and postnatal mothers attending reproductive and child health clinics in rural Zambia.

## 2. Materials and Methods

### 2.1. Study Design, Place of Study, and Participants

This was a quantitative, descriptive, cross-sectional health facility-based study. It was conducted in Chibombo and Chisamba rural districts located in the Central Province in Zambia. Five different health facilities participated including four rural health centres (RHC) and one district hospital. These health facilities were readily accessible and thus conveniently selected. Specific health facilities that participated were Chibombo RHC, Twalumba RHC, and Mwachisompola RHC from Chibombo district together with Malombe RHC and Liteta District Hospital from Chisamba district. Recruitment of study participants involved all pregnant women (PW) who were routinely attending 2nd visit antenatal clinic and all postnatal mothers (PM) who were routinely attending postnatal services provided that they were willing and thus gave their consent.

### 2.2. Sampling

The single stage cluster sampling was utilized to select the five RHC clinics for the study, and the recruitment of study participants was done by registering consecutively every consenting pregnant woman and postnatal mother in the selected health facilities until achieving the required sample size. No random sampling was undertaken rather a convenience sampling approach was used within the clinic setting.

### 2.3. Data Sources and Collection Procedure

A pretested and validated questionnaire which was prepared in English and translated in Bemba and Lenje, the local languages of the study participants, was used to interview 90% of the participants who could not read and write, and the rest undertook a self-administered questionnaire.

The data collection tool consisted of questions on sociodemographic factors (age, level of education, and marital status), knowledge of periodontal diseases (17 items), oral hygiene practices (9 items), and self-reported periodontal problems (5 items). Correct responses/answers in each section were summed up and divided over the total items for calculation of percentages out of 100% as regards to knowledge of periodontal diseases, oral hygiene practices, and self-reported periodontal problems. The respondents who scored above 50% were graded as “good” and those who scored below 50% were graded as “poor.”

### 2.4. Data Analysis

Data were entered into a computer and analyzed using Statistical Package for Social Sciences (SPSS) version 19.0. Frequency tables were generated. Data transformation was undertaken in particular dichotomization of some variables that had more than two options for example, age (15–24 years versus 25 years and above) and level of education (primary education and lower versus secondary, college, and university education). Furthermore, the type of occupation of the study participants was dichotomized and recoded as informal (none and self-employed) versus formal (employed and business persons), and health facility was dichotomized as hospital versus health centres. The responses to specific oral hygiene practices and self-reported periodontal problems were dichotomized into presence (Yes) or absence (No) of the specific condition. Cross tabulations were processed between dichotomized categorical variables that generated two-by-two contingency tables. The chi-square test or Fisher's exact test (in cross tabulation cases where one or more of the cells had a value of “5” or less) was used to detect statistically significant differences between two groups of any categorical variables under consideration. In all analyses, the statistical significance level was set at “<0.05.”

For binary logistic regression analyses, the dichotomized variables were recoded into zero (0) for a code that was assigned to an advantageous or nonproblematic aspect, and a code number of one (1) was given to any previous code number that meant to be in a disadvantageous or problematic aspect. For example, someone who is brushing the teeth twice a day in line with the international recommendation (thus being on an advantageous aspect) were given a code number of zero (0), and anyone who was not brushing according to this recommendation did put him/herself into disadvantageous aspect and thus was given a new code number of one (1). Also, anyone having a sign of a disease or condition under study was given a code number of one (1), whereas a study participant without any sign of that disease or condition was given a code number of zero (0). All those variables that were recoded for the binary logistic regression analyses were subjected to descriptive statistics in particular cross tabulation. Variables with a significant probability (*P*) value (*P* < 0.05) during cross tabulation were selected and entered into the logistic regression analysis. In addition, all variables that were thought to be important as predisposing factors for the dependent variable (gum bleeding) provided that *P* value was less than 0.3 (arbitrarily chosen) and were included in the logistic regression analyses. The characteristic of maternal status, meaning that the study participant was either “pregnant” or a “postnatal” woman, was deliberately included in the model although the probability value was far above the selected cutoff point because it was strongly felt that this factor is associated with bleeding gums. The backward stepwise (Wald) logistic regression method was chosen for analysis. The dependent variable was the self-reported condition as experienced “gum bleeding”' and the rest of the variables on oral hygiene practices, knowledge, and other predisposing conditions were considered as categorical “covariates” whereby the contrast was the “indicator” and reference category was set as the “first” category. The options were set at the 95% confidence interval (95% CI) for the exponential (B) whereby the probability for the backward stepwise (Wald) model was set at entry value of 0.05 and removal at value 0.10. The final iteration of the backward stepwise (Wald) model was included as final results.

## 3. Results

### 3.1. Distribution of Study Participants

A total of 410 study participants comprising 270 pregnant women and 140 postnatal mothers were recruited (Table 1) with age ranging from 15 to 43 years (Mean age ± standard deviation: 25.72 ± 6.88 years). The median age was 24 years. Majority of the study participants were at 15–24 years of age (65.7%). The ever married women were the majority (83%). Most of the study participants had primary education or less, and generally the employed group was 56%. Health centres had higher number of study participants (75%) as compared to the hospital.

### 3.2. Oral Hygiene Practices

The prevalence of oral hygiene practices and self-reported periodontal problems among pregnant women and postnatal mothers is shown in Table 2. All participants claimed to practice regular tooth brushing, but frequency of tooth brushing varied among individuals. Tooth brushing once per day was 24.9% and brushing twice per day was 38.5%. Those who brushed three times per day were 36.3%. Duration of tooth brushing was estimated to take about 1–3 minutes (57.1%). Chewing stick users were at 12.2%. Use of a plastic toothbrush to clean teeth (Table 2) was significantly higher among postnatal mothers (100%) than in pregnant women (93.3%, *P*=0.001, *χ*^2^ = 9.672).

Replacement of tooth brushes once/month was done by 25.4% while 39.3% replaced their toothbrushes after every three months. Slightly more than half of the study participants (55.4%) had the habit of cleaning the tongue regularly. Regular use of toothpaste during tooth brushing was reported by 91.7%, and there were no significant differences between pregnant women and postnatal mothers (Table 2).

Among all the study participants (*n*=410) who responded to the question whether they had ever heard of the gum or periodontal diseases, 254 (62%) participants answered correctly (Yes), and there were more pregnant women (179/270 (66.3%)) than postnatal mothers (75/140 (53.6%)) (*χ*^2^ = 6.333, *P*=0.012). Among all respondents (*n*=256), 222 (86.7%) gave the correct response to the question that gum or periodontal diseases can present itself in a form of gingival swelling, and there were more postnatal mothers (72/75 (96.0%)) than pregnant women (150/181 (82.9%)) (Fisher's exact test, *P*=0.004). Correct response to the question that gum or periodontal diseases can be prevented by visiting a dentist was given by 207/256 (80.9%) respondents, and there were more postnatal mothers (67/75 (89.3%)) than pregnant women (140/181 (77.3%)) (*χ*^2^ = 4.922, *P*=0.027). Eating balanced diet was considered to be one of the preventive measures for gum or periodontal diseases by 138/257 (53.7%) study participants where the proportion of postnatal mothers was higher (49/76 (64.5%)) than pregnant women (89/181 (49.2%)) (*χ*^2^ = 5.041, *P*=0.025). Use of plastic toothbrush was significantly higher among postnatal mothers (100%) than pregnant women (93.3%) (*χ*^2^ = 9.762, *P*=0.001). Chewing stick users were almost equally distributed among the pregnant women (11.1%) and postnatal mothers (10.0%) (*χ*^2^ = 0.119, *P*=0.865). Those who opted to use a finger for teeth cleaning were only found among pregnant women (3/270 (1.1%)); there were none from the group of postnatal mothers, and the differences were not statistically significant (Table 2). Two out of three (66.7%) participants who used a finger for teeth cleaning also reported to use toothpaste.

### 3.3. Self-Reported Periodontal Problems

The prevalence of self-reported periodontal problems included bleeding gums (23.2%), painful gums (15.9%), swollen gums (11.0%), reddish gums (10.5%), and tooth mobility (3.4%), and there was no statistically significant difference between pregnant women and postnatal mothers (Table 2). The differences in the proportion of study participants who have heard about dental plaque among pregnant women (184/270 (68.1%)) versus postnatal mothers (96/140 (68.6%)) as well as about calculus (171/270 (63.3%)) versus (81/140 (57.9%)), respectively, were not statistically significant (table not shown).

The level of knowledge of oral hygiene practices when categorized as “good” or “poor” in relation to different demographic factors among women attending the RHC clinics in rural Zambia is shown in Table 3.

Of all the participants in the category of good knowledge, there were more married women than singles, more of the low level of education, and more from the health centres than their respective counterparts. However, the level of knowledge of oral hygiene practices (good versus poor) did not statistically differ significantly between age groups, marital status, level of education, employment, and type of health facility attended. There was a significantly higher proportion of pregnant women (Table 3) in the category of poor knowledge (69.8%) as compared to that of good knowledge of oral hygiene practices (59.2%) (*χ*^2^ = 4.741, *P*=0.029) (Table 3).

The level of knowledge of gum and periodontal diseases in relation to different demographic factors among women attending the RHC clinics in rural Zambia is shown in Table 4. The level of knowledge of gum and periodontal diseases as categorized as “good” or “poor” in various demographic factors including pregnant women and postnatal mothers, low and high education, singles and married women, and employment status was homogeneous in that the difference was not statistically significant (Table 4).

Results of the bivariate analysis regarding self-reported gum bleeding in relation to maternal status (pregnant woman or postnatal mother), sociodemographic factors, lack of knowledge on periodontal diseases, oral hygiene practices, and self-reported periodontal problems are shown in Table 5. Prevalence of self-reported gum bleeding was significantly associated with being of older age 25–45 years (*χ*^2^ = 4.04, *P*=0.036) and lack of knowledge that eating balanced diet can prevent gum and periodontal diseases (*χ*^2^ = 5.527, *P*=0.019). Other significant factors were as follows: not changing the toothbrush after a period of 1–3 months (*χ*^2^ = 10.766, *P*=0.001), self-reported presence of swollen gums (*χ*^2^ = 97.703, *P* < 0.001), reddish gums (*χ*^2^ = 70.871, *P* < 0.001), painful gums (*χ*^2^ = 98.310, *P* < 0.001), and shaky (mobile) teeth (*χ*^2^ = 18.812, *P* < 0.001).

The final model of the binary logistic regression analyses (backward stepwise, Wald) for occurrence of self-reported gum bleeding in relation to selected demographic factors and periodontal problems among pregnant women and postnatal mothers is shown in Table 6.

Factors that more likely and significantly associated with self-reported gum bleeding were being a pregnant woman (odds ratio (OR): 6.198, 95% confidence interval (CI): 1.620–23.715, *P*=0.008), presence of reddish gums (OR: 4.724, 95% CI: 1.375–16.225, *P*=0.014), painful gums (OR: 21.901, 95% CI: 6.731–71.264, *P* < 0.001), and toothpick use (OR: 4.288, 95% CI: 1.110–16.571, *P*=0.035).

## 4. Discussion

The study was a quantitative, descriptive, cross-sectional investigation that took place among pregnant women and postnatal mothers in Chibombo and Chisamba rural districts in Zambia. The sample size and age ranges were almost similar to a study done in Nigeria [9]. This could be due to the fact that this is the reproductive age (15–49 years) in sub-Saharan Africa [17]. In the current study, it showed that majority of the women did not go to school or went up to primary level. This could be due to poverty and early marriages which stands at 31.4% in Zambia [18], especially in rural areas where this study was conducted as well as long distances to a few available schools [19] which led many to drop out of school and end up getting married.

The proportion of study participants that had primary education was similar to a study done in Pakistan [20], where more than half of the participants ended at primary school level (Asia levels at 64% versus sub-Saharan Africa with 65% [21]). The current study found that more than three quarters were married (83%) than the singles. This is different from the study done in south-west Sydney [22], where more than half of the participants were single, and it is speculated that the possible reason might be the difference in culture and lifestyles. Health centres had more RHC clinic attendances than the hospital. This can be attributed to the number of rural health centres included in the study (four) versus one rural hospital as is typical of levels and referral health system in Zambia. Another study done in Nigeria [9] reported that women do not seek professional help if they perceive that their gingival status is normal and that women were more likely to use dental services in pregnancy if married, educated, and had dental insurance. However, for comparability, retrievable reports on oral health among pregnant women in Zambia were scarce.

In the current study, almost all the respondents were brushing their teeth at least once per day and the finding is consistent with what was reported elsewhere in Tanzania [23]. The use of chewing sticks (twigs or roots of certain plants that are chewed until one end is frayed and used to clean teeth) in the current study was slightly more than one to ten (1 : 10), and the possible explanation might be due to difficult affordability within the rural constraint economy and these results are consistent with the studies done in Nigeria and Tanzania, respectively [2, 23]. In the present study, a minority of the subjects use dental floss, unlike the study done in Australia [6] where the majority were using dental floss with an understanding that it would help prevent gum disease. This shows that the participants in the current study in rural Zambia had insufficient knowledge on interdental space cleaning and were limited to toothbrushes as cleaning aids [24]. Most of the study participants pointed out that plaque can be controlled by maintaining good oral hygiene, and this is achieved by brushing the teeth at least twice daily and this is in line with what is recommended worldwide [21]. Serious attention to this important preliminary understanding emphasis on oral hygiene instruction, for example, systematic tooth brushing for two minutes [25] and interdental cleaning might be a good area to begin with when launching customized oral health program in Zambia. The most used tooth cleaning aids were plastic toothbrush, followed by chewing stick, whereby the latter is believed to be an effective oral hygiene aid by which different cultures have attached functional value since ancient times [26]. It happened that about one percent of the pregnant women used a finger for cleaning teeth whereas their level of education was above primary school. This could be due to extreme poverty as most of them were unemployed. Also, the issue of beliefs cannot be underestimated because these participants, for example, could have used chewing sticks that were readily available in rural areas.

The proportion of knowledge of periodontal diseases displayed by women in the age group less than 30 years was moderately higher compared to those above 30 years, and further, it was higher among the singles as compared to the married; however, the differences did not reach a statistically significant level. The study participants who had attained secondary education were more knowledgeable than the ones who were primary school leavers or below. This simply shows that education plays a part in terms of knowledge and exposure [27]; however, the difference was statistically insignificant. The proportion of pregnant women that had knowledge of periodontal disease was moderately higher than the postnatal mothers. The reason could be that pregnant women were able to identify themselves with the features of periodontal diseases that are modified by the presence of high levels of circulating hormones during pregnancy; however, the differences did not reach a statistically significant level. Even though the pregnant women and the postnatal mothers were knowledgeable about periodontal diseases, only a minority were aware of the causative factor and dental plaque. This may point to a serious need for proper oral health education to the pregnant women and postnatal mothers in the studied rural population. Slightly less than three quarters of the participants knew what plaque was and how it could be removed, and this is consistent with the Saudi Arabia study [28], but inconsistent with the findings from elsewhere [6] where the majority knew about dental plaque and did not know about periodontal disease. The possible explanation for this might be the differences in the availability of oral health education and health promotion programs in these populations [29]. The majority of the pregnant women and postnatal mothers knew the presentation of periodontal disease as well as the prevention. Proper nutrition and healthy lifestyle also play a key role in the general well-being of the mother to be, and this includes periodontal health [30].

Regarding self-reported gum and periodontal problems, a minority of the study participants reported having bleeding gums, and the findings are similar to those reported by women attending a tertiary health institution in Nigeria [31]. On the other hand, our findings differ from the ones reported in Nigerian women [32] and Ghanaian women [33] where bleeding of gums was much higher than what was found in our study, and the most probable explanation is the difference in methodology. The current study has a low proportion of women reporting painful gums, swollen gums, and bleeding gums as compared to other studies which revealed that hormonal changes in pregnancy combined with neglected oral hygiene tend to increase the gingivitis which is characterized by increased redness, edema, and higher tendency toward bleeding [34, 35].

Use of plastic toothbrush in our study was significantly higher among pregnant mothers than postnatal women, and this difference might be accounted by possible exposure to oral health education session during antenatal visits. The results in the current study are similar to the study done in Nigeria [36] where most of the participants used plastic toothbrushes and paste.

Likewise, there were no statistically significant differences on the level of knowledge of gum and periodontal diseases in relation to different demographic factors between pregnant women and postnatal mothers in our study, thus showing a similar experience between the groups. These results are in agreement with Bangalore report where awareness of gum disease among pregnant women was not associated with age and educational qualifications [37].

Lack of knowledge that eating balanced diet can prevent gum and periodontal diseases was in the bivariate analysis found to be a significant factor as regards to the self-reported gum bleeding on tooth brushing. Lack of knowledge on the importance of balanced diet was higher in our study than in Bilaspur, India [38], and the possible reason among others was that our study was done in rural area alone while the latter was in both urban and rural. In comparison with postnatal mothers, pregnant women were significantly more likely to experience gum bleeding on tooth brushing, and this might be explained by the inflammatory reaction of the gingival due to hormonal changes coupled with presence of poor oral hygiene [34, 35]. Findings from a similar study in India revealed that less than one third of the studied pregnant women had experienced bleeding from gums during pregnancy, and that, slightly less than a quarter did not brush their teeth when they experienced bleeding, instead, they cleaned using fingers [39].

The results of this study must be viewed in the light of certain limitations. Due to constraint in resources especially time and funds to collect data for this elective study, the rural area was selected for convenience. This approach limits the inference of the findings to be much more applicable to the rural districts studied population and not to the whole RHC clinic attendees in the country. This study relied on self-reported information and therefore the data are subject to some form of bias. Furthermore, the face-to-face interview with most of the study participants might have provoked “socially desirable responses” instead of what was the real practice in daily life [40, 41].

## 5. Conclusions

In this study, most pregnant women and postnatal mothers had general knowledge of periodontal diseases but only few knew the cause and their prevention. All participants were engaged in tooth cleaning procedures; however, the majority did not know the appropriate practices. Self-reported signs of gingival and periodontal diseases were experienced by the minority.

## 6. Recommendations

In view of the ever growing evidence that periodontal diseases are associated with various systemic conditions including adverse pregnancy outcomes, it is recommended that oral health be integrated into general health care of pregnant women in all reproductive and child health clinics in the country.

## Figures and Tables

**Table 1:** Distribution of the study participants by demographic characteristics.

Sociodemographic characteristics	Pregnant women, *n* (%)	Postnatal mothers, *n* (%)	All (*n*=410), *n* (%)	*χ* ^2^ value	*P* value
Age group					
15–24 years	136 (50.4)	71 (50.7)	207 (50.5)	0.004	0.947
25–45 years	134 (49.6)	69 (49.3)	203 (49.5)		

Education level					
No/primary education	163 (60.4)	75 (53.6)	238 (58.0)	1.750	0.186
Secondary and above	107 (39.6)	65 (46.4)	172 (42.0)		

Marital status					
Single	47 (17.4)	22 (15.7)	69 (16.8)	0.189	0.664
Ever married	223 (82.6)	118 (84.3)	341 (83.2)		

Employment					
Unemployed	113 (41.9)	69 (49.3)	182 (44.4)	2.064	0.151
Employed	157 (58.1)	71 (50.7)	228 (55.6)		

Health facility					
Hospital	60 (22.2)	40 (28.6)	100 (24.4)	2.015	0.156
Health centres	210 (77.8)	100 (71.4)	310 (75.6)		

**Table 2:** Oral hygiene practices and self-reported periodontal problems among pregnant women and postnatal mothers in rural Zambia.

Oral hygiene practices	Distribution in percentages (%)			*χ* ^2^ value	*P* value
	All (*n*=410)	Pregnant women (*n*=270)	Postnatal mothers (*n*=140)		
Cleaning of teeth and gums	98.3	98.9	97.1	1.675	0.196
Brushing once a day	24.9	23.0	28.6	3.798	0.284
Brushing twice a day	38.5	37.4	40.7	3.798	0.284
Brushing three times a day	36.3	39.3	30.7	3.798	0.284
Brushing once a week	0.2	0.4	0.0	3.798	0.284
Brush less than 1 minute	25.9	26.3	25.0	3.231	0.199
Brush 1–3 minutes	57.1	54.4	62.1	3.231	0.199
Brush more than 3 minutes	17.1	19.3	12.9	3.231	0.199
Use of toothbrush to clean teeth	95.6	93.3	100	9.762	0.001
Use of chewing stick to clean teeth	12.2	11.1	10.0	0.119	0.865
Use of toothpick to clean teeth	10.7	13.0	10.7	0.435	0.509
Use of finger to clean teeth	0.7	1.1	0.0	1.567	0.554
Regular use of toothpaste	91.7	92.2	90.7	0.276	0.600
Tongue brushing	55.4	53.3	59.3	1.322	0.250
Flossing	2.0	2.2	1.4	#	0.721
Use of mouth wash	13.9	16.3	9.3	3.786	0.070
Changes toothbrush (TBR) once/month	25.4	27.4	21.4	6.002	0.199
Changes TBR after 3 months	39.3	37.4	42.9	6.002	0.199
Changes TBR after 1 year	6.8	6.7	7.1	6.002	0.199
Changes TBR when bristles bend	25.6	24.4	27.9	6.002	0.199
Changes not the TBR	2.9	4.1	0.7	6.002	0.199
Bleeding gums	23.2	23.3	22.9	0.012	0.914
Painful gums	15.9	14.8	17.9	0.640	0.424
Swollen gums	11.0	10.7	11.4	0.45	0.833
Reddish gums	10.5	10.4	10.7	0.012	0.914
Tooth mobility	3.4	3.7	2.9	0.212	0.645

^#^Fisher's exact test (no chi-square value as the chi-square test was not used for this item) as one cell had 2 study participants only that is less than the minimum of 5 subjects.

**Table 3:** The level of knowledge of oral hygiene practices in different demographic factors among women attending the RHC clinics in rural Zambia.

Demographic factors	Level of knowledge of oral hygiene practices		*χ* ^2^ value	*P* value
	Good *n* (%)	Poor *n* (%)		
Age group				
15–24 years	75 (49.3)	132 (51.2)	0.127	0.722
25–45 years	77 (50.7)	126 (48.8)		

Marital status				
Single	23 (15.1)	46 (17.8)	0.497	0.481
Ever married	129 (84.9)	212 (82.2)		

Education level				
No/primary education	86 (56.6)	152 (58.9)	0.214	0.643
Secondary and above	66 (43.4)	106 (41.1)		

Employment				
Unemployed	71 (46.7)	111 (43.0)	0.527	0.468
Employed	81 (53.3)	147 (57.0)		

Health facility				
Hospital	44 (28.9)	56 (21.7)	2.720	0.099
Health centres	108 (71.1)	202 (78.3)		

Study participants				
Pregnant women	90 (59.2)	180 (69.8)	4.741	0.029
Postnatal mothers	62 (40.8)	78 (30.2)		

**Table 4:** The level of knowledge of gum and periodontal diseases in relation to different demographic factors among women attending the RHC clinics in rural Zambia.

Demographic factors	Level of knowledge of gum/periodontal disease		*χ* ^2^ value	*P* value
	Good *n* (%)	Poor *n* (%)		
Women attending RHC				
Pregnant women	116 (69.5)	90 (61.6)	2.116	0.146
Postnatal mothers	51 (30.5)	56 (38.4)		

Age group				
15–24 years	87 (52.1)	65 (44.5)	1.790	0.181
25–45 years	80 (47.9)	81 (55.5)		

Education level				
No/primary education	92 (55.1)	85 (58.2)	0.310	0.577
Secondary and above	75 (44.9)	61 (41.8)		

Marital status				
Single	30 (18.0)	23 (15.8)	0.271	0.603
Ever married	137 (82.0)	123 (84.2)		

Employment				
Unemployed	73 (43.7)	67 (45.9)	0.149	0.699
Employed	94 (56.3)	79 (54.1)		

Health facility				
Hospital	45 (26.9)	36 (24.7)	0.213	0.645
Health centres	122 (73.1)	110 (75.3)		

**Table 5:** Bivariate analysis: self-reported gum bleeding in relation to sociodemographic factors, knowledge, oral hygiene practices, and self-assessed periodontal status.

Characteristics of the study participants	Whole sample (*n*=410)		Self-reported gum bleeding				*χ* ^2^ value	*P* value
			Yes		No			
	*n*	%	*n*	%	*N*	%		
Maternal status								
Had pregnancy	270	65.9	63	66.3	207	65.7	0.012	0.914

Sociodemographic factors								
Age 25–45 years (not 15–24 years)	203	49.5	56	58.9	147	46.7	4.404	0.036
Had primary education or less	238	58.0	62	65.3	176	55.9	2.643	0.104
Not employed or have petty business	302	73.7	64	67.4	238	75.6	2.521	0.112

Knowledge: lack of knowledge								
Have not heard about plaque	130	31.7	35	36.8	95	30.2	1.506	0.220
Have not heard about calculus	158	38.1	41	43.2	117	37.1	1.115	0.291
On causes of periodontal diseases	146	46.6	37	52.1	109	45.0	1.103	0.294
That PD presents with gum bleeding	19	7.4	2	3.4	17	8.6	1.723	0.189
That PD presents with gum swelling	34	13.3	5	8.6	29	14.6	1.414	0.234
That PD presents with reddish gums	39	15.2	5	8.6	34	17.2	2.540	0.111
That calculus can be removed	107	42.5	27	50.0	80	40.4	1.599	0.206
That good oral hygiene can prevent PD	37	14.6	5	8.8	32	16.2	1.983	0.159
That eating balanced diet can prevent PD	119	46.3	19	32.8	100	50.3	5.527	0.019
That visiting a dentist can prevent PD	49	19.1	8	13.8	41	20.7	1.386	0.239

Oral hygiene practices								
Not using plastic toothbrush	18	4.4	7	7.4	11	3.5	2.613	0.106
Not changing toothbrush 1–3 months	145	35.4	47	49.5	98	31.1	10.766	0.001
Uses toothpick	50	12.2	17	17.9	33	10.5	3.751	0.053

Self-reported periodontal problems								
Had swollen gums	45	11.0	36	37.9	9	2.9	91.703	<0.001
Had reddish gums	43	10.5	32	33.7	11	3.5	70.871	<0.001
Had painful gums	65	15.9	46	48.4	19	6.0	98.310	<0.001
Had shaky teeth	14	3.4	10	10.5	4	1.3	18.812	<0.001

^#^Each condition presented in this table has basically ““Yes and No” alternatives with numerical values corresponding to each individual situation. Only the numerical values corresponding to “Yes” have been presented in this table and the counterpart alternative (“No”) numerical values have been left out. For example, if have swollen gums (“Yes versus No”), only the numerical values for “Yes” have been presented in this table while the ones corresponding to “No” have been left out; PD = periodontal diseases.

**Table 6:** Final model of logistic regression backward stepwise (Wald) analyses: binary logistic regression analyses in relation to self-reported gum bleeding versus demographic factors and periodontal problems among the study participants.

Characteristics of the study participant	*B*	SE	Odds ratio	95% confidence interval	*P* value
Had pregnancy	1.824	0.685	6.198	1.620–23.715	0.008
Had reddish gums	1.553	0.630	4.724	1.375–16.225	0.014
Had gum pains	3.087	0.602	21.901	6.731–71.264	<0.001
Uses toothpicks	1.456	0.690	4.288	1.110–16.571	0.035

Key: *B* = beta weights (regression coefficient), SE = standard error.

## Data Availability

Quantitative data were used. Data used to support the findings of this study are available from the corresponding author upon request.

## References

[1] Armitage G.C. (1999). Development of a classification system for periodontal diseases and conditions. *Annals of Periodontology*.

[2] Shaw L., Harjunmaa U., Doyle R. (2016). Distinguishing the signals of gingivitis and periodontitis in supragingival plaque, a cross-sectional study in Malawi. *Applied and Environmental Microbiology*.

[3] De Franceschi M.S., Fortunato L., Carallo C. (2012). Periodontal disease and carotid atherosclerosis: mechanisms of the association. *Oral Health Care-Prosthodontics, Periodontology, Biology, Research and Systemic Conditions*.

[4] Silva N., Abusleme L., Bravo D. (2015). Host response mechanisms in periodontal diseases. *Journal of Applied Oral Science*.

[5] Carillo-de-Albornoz A. F. E., Herrera D. C. P., Bascones-Martinez A. (2011). Gingival changes during pregnancy: III. Impact of clinical, microbiological, immunological and socio-demographic factors on gingival inflammation. *Journal of Clinical Periodontology*.

[6] Thomas N. J., Middleton P. F., Crowther C. A. (2008). Oral and dental health care practices in pregnant women in Australia: a postnatal survey. *BMC Pregnancy and Childbirth*.

[7] Ovadia R., Zirdok R., Diaz-Romero R. M. (2007). Pregnancy outcomes influenced by periodontitis. *Medicine and Biology*.

[8] Otomo-Corgel J., Carranza F. A., Klokkevold P. R., Takei H. H., Newman M. J. (2012). Periodontal therapy in the female patient. *Carranza’s Clinical Periodontology*.

[9] Ifesanya J. U., Oke G. A. (2013). Self-report of adverse gingival conditions among pregnant south-western Nigerian women. *Journal of Dentistry and Oral Hygiene*.

[10] Geisinger M. L., Robinson M., Kaur M. (2013). Individualized oral health education improves oral hygiene compliance and clinical outcomes in pregnant women with Gingivitis. *Journal of Oral Hygiene and Health*.

[11] Srivastava A., Gupta K. K., Srivastava S., Garg J. (2011). Effects of sex hormones on the gingiva in pregnancy: a review and report of two cases. *Journal of Periodontology and Implant Dentistry*.

[12] Villa A., Abati S., Strohmenger L., Cargnel M., Cetin I. (2011). Self-reported oral hygiene habits and periodontal symptoms among postpartum women. *Archives of Gynecology and Obstetrics*.

[13] Xiong X., Buekens P., Fraser W. D., Beck J., Offenbacher S. (2006). Periodontal disease and adverse pregnancy outcomes: a systematic review. *An International Journal of Obstetrics and Gynaecology*.

[14] Silness J., Loe H. (1964). Periodontal disease in pregnancy II. Correlation between oral hygiene and periodontal condition. *Journal Acta Odontologica Scandinavica*.

[15] Laine M. A. (2002). Effects of pregnancy on periodontal and dental health. *Journal Acta Odontologica Scandinavica*.

[16] Barness C. M., Russel C. M., Reinhardt R. A., Payne J. B., Lyle D. M. (2005). Comparison of irrigation to floss as an adjunct to tooth brushing: effects on bleeding, gingivitis and supragingival plaque. *Journal of Clinical Dentistry*.

[17] Ronsmans C., Graham W. J. (2006). Maternal mortality, who, when, where and why. *The Lancet*.

[18] UNFPA and Government of the Republic of Zambia (2017). *Policy Brief, Child Marriage in Zambia, Lusaka, Zambia*.

[19] UNICEF ESARO and UIS (2014). Global initiative on out-of-school children.

[20] Shabbir S., Zahi M., Qazi A. (2015). Oral hygiene among pregnant women, practices and knowledge. *Professional Medical Journal*.

[21] Zhu L., Peterson P.E., Wang H., Bian J., Zhang B. (2005). Oral health knowledge attitude and behavior of adults in China. *International Dental Journal*.

[22] George A., Johnson M., Blinkhorn A. (2013). The oral health status, practices and knowledge of pregnant women in south-west Sydney. *Australian Dental Journal*.

[23] Mumghamba E. G., Manji K. P., Michael J. (2006). Oral health practices, periodontal conditions status and self-reported bad mouth breath among mothers, Tanzania. *International Journal of Dental Hygiene*.

[24] Ramamurthy J., Irfana F. (2017). Assessment of knowledge and awareness about periodontal oral health among pregnant women-a questionnaire study. *International Journal of Current Pharmaceutical Review and Research*.

[25] Asadoorian J. (2006). CDHA position paper on tooth brushing. *Canadian Journal of Dental Hygiene*.

[26] Nordin F. N. M., Mohsain S. R. A. S., Tamizi S. M. (2012). A review on the Sunnah of Miswak (Salvadora Persica) and its potentiality to improve oral health. *Revelation and Science*.

[27] Duha A., Colin C., Naci M. (2011). The impact of education on health knowledge. *Economics of Education Review*.

[28] Asaad F. A., Rahman G. A. I., Mahmoud N. A. I., Shamasi E., Alkhuwailerdj A. (2015). Periodontal disease awareness among pregnant women in the central eastern region of Saudi Arabia. *Journal of Investigative and Clinical Dentistry*.

[29] Nakre P. D., Harikiran A. G. (2013). Effectiveness of oral hygiene education progress: a systematic review. *Journal of International Society of Preventive and Community Dentistry*.

[30] Najeeb S., Zafar M. S., Khurshid Z., Zohaib S., Almas K. (2016). The role of nutrition in periodontal health: an update. *NCBI Resources*.

[31] Bishiru B. O., Anthony I. N. (2014). Oral health awareness and experience among pregnant women in Nigerian Tertiary Health Institution. *Journal of Dental Research and Review*.

[32] Onigbinde O. O., Sorunke M. E., Braimoh M. O., Adeniyi A. O. (2014). Periodontal status and some variables among pregnant women in a Nigeria Tertiary Institution. *Annals of Medical and Health Sciences Research*.

[33] Nuamah I., Annan B. D. (1998). Periodontal status and oral hygiene practices of pregnant and non-pregnant women. *East African Medical Journal*.

[34] Hasan M. R., Dithi A. B., Nomann N. A., Nessa J., Saito T. (2015). Self-reported oral and dental health status among pregnant women of a selected hospital in Dhaka city. *Bangladesh Journal of Dental Research and Education*.

[35] Chandropooja J., Gayathri R., vishnupri V. (2016). Oral health during pregnancy-a systematic review. *Journal of Pharmaceutical Sciences and Research*.

[36] Ifesanya J. U., Ifesanya A. O., Asuzu M. C., Oke G. A. (2010). Determinants of good oral hygiene among pregnant women in Ibadan, south-western Nigeria. *Annals of Ibadan Postgraduate Medicine*.

[37] Singh S., Dagrus K., Kariya P. B., Singh S., Darmina J., Hase P. (2015). Oral periodontal health knowledge and awareness among pregnant females in Bangalore, India. *International Journal of Dental and Medical Research*.

[38] Nagi R., Sahu S., Nagaraju R. (2016). Oral health, nutritional knowledge, and practices among pregnant women and their awareness relating to adverse pregnancy outcomes. *Journal of Indian Academy of Oral Medicine and Radiology*.

[39] Sajjan P., Pattanshetti J. I., Padmini C., Nagathan V. M., Sajjanar M., Siddiqui T. (2015). Oral health related awareness and practices among pregnant women in Bagalkot district, Karnataka, India. *Journal of International Oral Health*.

[40] Abramson J. H., Abramson Z. H. (1999). *Survey Methods in Community Medicine, Epidemiological Research, Program Evaluation and Clinical Trials*.

[41] Sanzone L. A., Lee J. Y., Divaris K., DeWalt D. A., Baker A. D., Vann W. F. (2013). A cross sectional study examining social desirability bias in caregiver reporting of children’s oral health behaviors. *BMC Oral Health*.

